# The genome sequence of the Feathered Ranunculus, *Polymixis lichenea *(Hübner, 1813)

**DOI:** 10.12688/wellcomeopenres.20007.1

**Published:** 2023-10-12

**Authors:** David C. Lees, Natural History Museum Genome Acquisition Lab, Darwin Tree of Life Barcoding collective, Wellcome Sanger Institute Tree of Life programme, Wellcome Sanger Institute Scientific Operations: DNA Pipelines collective, Tree of Life Core Informatics collective, Darwin Tree of Life Consortium

**Affiliations:** 1 Natural History Museum, London, England, UK

**Keywords:** Polymixis lichenea, Feathered Ranunculus, genome sequence, chromosomal, Lepidoptera

## Abstract

We present a genome assembly from an individual male *Polymixis lichenea* (the Feathered Ranunculus; Arthropoda; Insecta; Lepidoptera; Noctuidae). The genome sequence is 716.7 megabases in span. Most of the assembly is scaffolded into 30 chromosomal pseudomolecules, including the Z sex chromosome. The mitochondrial genome has also been assembled and is 15.48 kilobases in length.

## Species taxonomy

Eukaryota; Metazoa; Eumetazoa; Bilateria; Protostomia; Ecdysozoa; Panarthropoda; Arthropoda; Mandibulata; Pancrustacea; Hexapoda; Insecta; Dicondylia; Pterygota; Neoptera; Endopterygota; Amphiesmenoptera; Lepidoptera; Glossata; Neolepidoptera; Heteroneura; Ditrysia; Obtectomera; Noctuoidea; Noctuidae; Xyleninae; *Polymixis*; *Polymixis lichenea* (Hübner, 1813) (NCBI:txid1870252).

## Background

The Feathered Ranunculus, *Polymixis lichenea* (Hübner, 1813) is a moderate sized noctuid moth with a wingspan of about 35–40 mm. Its forewings have a greenish tint, finely dotted with grey. The orbicular and reniform markings sometimes stand out with whitish margins or centres, as does occasionally a darker median fascia enveloping them; subterminal markings also include darker marks and other cross lines intermingle lighter and darker highlights. Local forms may be paler, darker, yellower or greener, apparently varying with substrate. Hindwings are dorsally darker in the female. The adult male, which tends to fly after midnight ( Skinner & Wilson, 2009), has pectinate antennae, as suggested by the vernacular name. The moth’s flight period is in the Palaearctic Autumn, in the UK from August to October ( Randle *et al.*, 2019), and it overwinters as a small larva ( Waring *et al.*, 2017).

The Feathered Ranunculus has a strong preference for coastal regions and cliffs as well as dunes, shingle and other sparsely vegetated habitats ( Waring *et al.*, 2017). The larva feeds on various low growing plants such as Biting Stonecrop ( *Sedum acre*), Thrift ( *Armeria maritima*), Ragwort ( *Senecio jacobaeae*), *Rumex* spp., Hoary Cress ( *Cardaria draba*), and *Brassica oleracea* ( Skinner & Wilson, 2009), but newly emerged larvae can be very fussy and will accept grass ( Leverton, 2001). The adult nectars on ivy blossom ( Skinner & Wilson, 2009).

*P. lichenea* is distributed towards coasts locally in the Western Palaearctic only, but absent in western Ireland and most of northern Britain ( Randle *et al.*, 2019), and ranges from north-east Ireland to the northern Baltic, Spain and Southern France but missing in central Europe and Scandinavia, with records in the Canaries and north-west Africa: Morocco ( GBIF Secretariat, 2022).

Populations in the UK have declined by 39% in abundance between 1970 and 2016 and by 21% in distribution ( Randle *et al.*, 2019). Between 1968 and 2006 Rothamsted trap numbers declined annually by an average of about 0.7% ( Conrad *et al.*, 2006).

*P. lichenea* exhibits a single mitochondrial cluster on BOLD, BOLD:AAH7735 (24/08/2023) with UK populations closer to those of Norway than the Mediterranean ones which are up to 1.38% divergent from them (maximum intraspecific divergence 1.77%). Several near neighbours of *P. lichenea* on BOLD belonging to different genera include species of *Mniotype* Franclemont, 1941, *Fishia* Grote, 1877 and *Dryotype* Hampson, 1906, whilst *Polymixis polymita* (Linnaeus, 1761) (the type species of *Polymixis* Hübner, [1820]) is at least 6.27% pairwise divergent to it in COI-5P (24/08/2023). *Polymixis* is a member of the noctuine tribe Xylenini, and ( Fibiger *et al.*, 2001) place *P. lichenea i*n the subgenus *Eumichtis* Hübner, 1821. Resolving the phylogenetic relationships of *Polymixis* will require multiple loci, which the genome sequence presented here will help to provide.

## Genome sequence report

**Figure 1.**
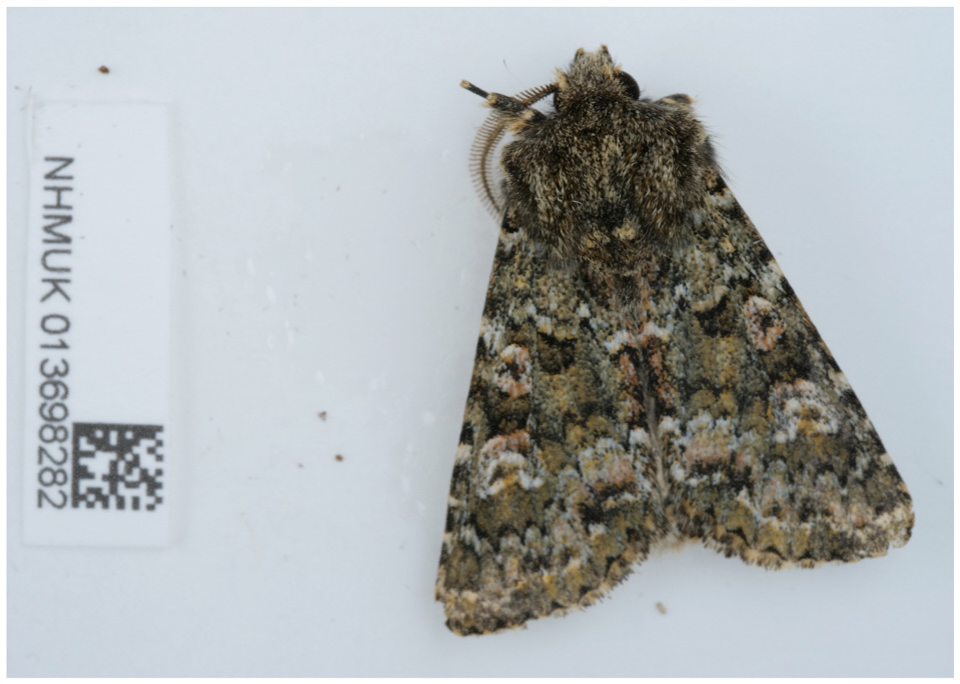
Photograph of the *Polymixis lichenea* (ilPolLich1) specimen used for genome sequencing.

The genome was sequenced from one male *Polymixis lichenea* ( Figure 1) collected from Restharrow Dunes National Nature Reserve, England, UK (51.27, 1.38). A total of 40-fold coverage in Pacific Biosciences single-molecule HiFi long reads was generated. Primary assembly contigs were scaffolded with chromosome conformation Hi-C data. Manual assembly curation corrected nine missing joins or mis-joins and removed two haplotypic duplications, reducing the scaffold number by 4.65%.

**Figure 2.**
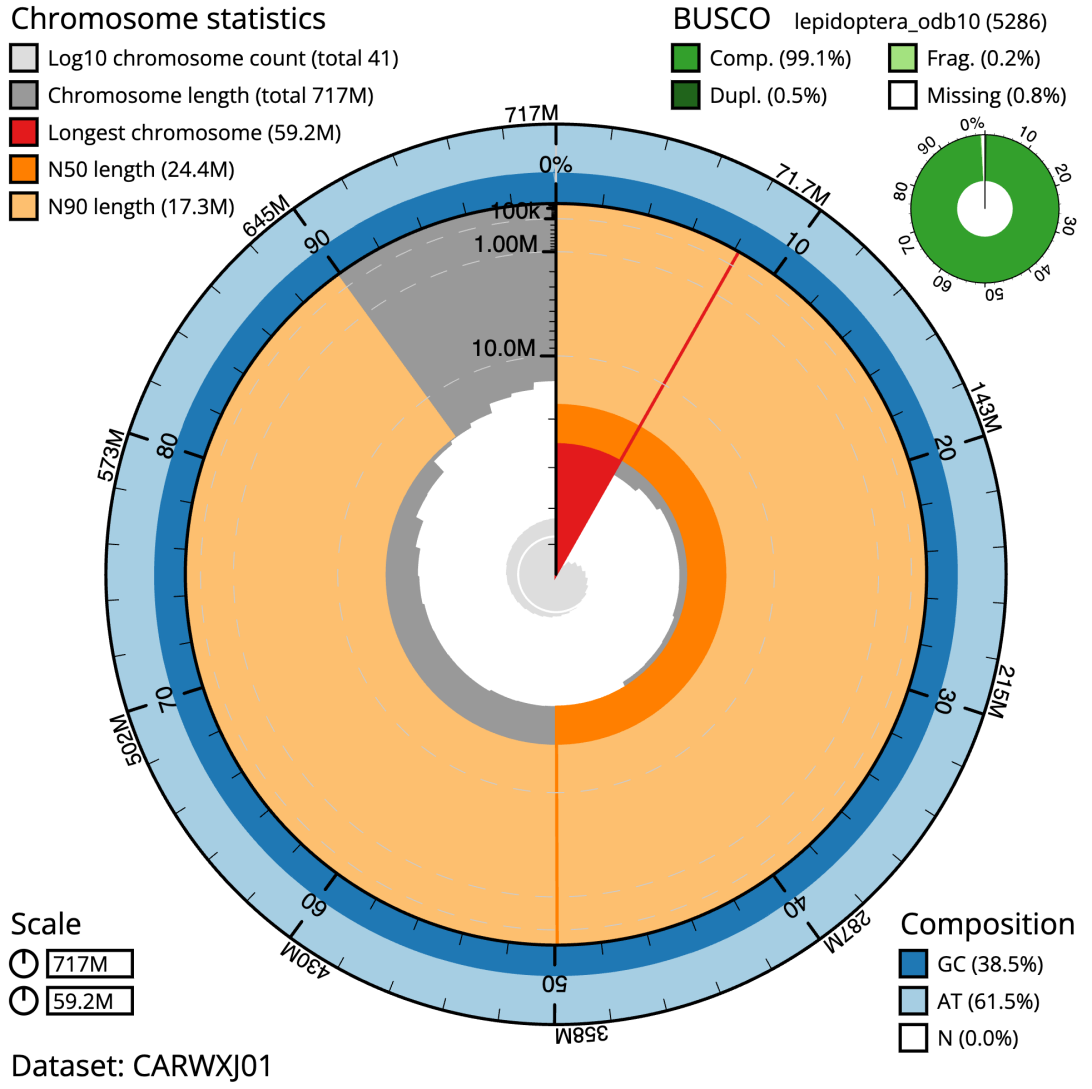
Genome assembly of *Polymixis lichenea*, ilPolLich1.1: metrics. The BlobToolKit Snailplot shows N50 metrics and BUSCO gene completeness. The main plot is divided into 1,000 size-ordered bins around the circumference with each bin representing 0.1% of the 716,694,765 bp assembly. The distribution of scaffold lengths is shown in dark grey with the plot radius scaled to the longest scaffold present in the assembly (59,173,276 bp, shown in red). Orange and pale-orange arcs show the N50 and N90 scaffold lengths (24,363,533 and 17,269,570 bp), respectively. The pale grey spiral shows the cumulative scaffold count on a log scale with white scale lines showing successive orders of magnitude. The blue and pale-blue area around the outside of the plot shows the distribution of GC, AT and N percentages in the same bins as the inner plot. A summary of complete, fragmented, duplicated and missing BUSCO genes in the lepidoptera_odb10 set is shown in the top right. An interactive version of this figure is available at https://blobtoolkit.genomehubs.org/view/ilPolLich1.1/dataset/CARWXJ01/snail.

The final assembly has a total length of 716.7 Mb in 40 sequence scaffolds with a scaffold N50 of 24.4 Mb ( Table 1). Most (99.94%) of the assembly sequence was assigned to 30 chromosomal-level scaffolds, representing 29 autosomes and the Z sex chromosome. Chromosome-scale scaffolds confirmed by the Hi-C data are named in order of size ( Figure 2– Figure 5; Table 2). Z chromosome identified based on synteny with *Fissipunctia ypsillon* (GCA_947568875.1) and *Dryobota labecula* (GCA_947523025.1). While not fully phased, the assembly deposited is of one haplotype. Contigs corresponding to the second haplotype have also been deposited. The mitochondrial genome was also assembled and can be found as a contig within the multifasta file of the genome submission.

**Table 1.** Genome data for *Polymixis lichenea*, ilPolLich1.1.

Project accession data		
Assembly identifier	ilPolLich1.1	
Species	*Polymixis lichenea*	
Specimen	ilPolLich1	
NCBI taxonomy ID	1870252	
BioProject	PRJEB59081	
BioSample ID	SAMEA111458575	
Isolate information	ilPolLich1: male (DNA sequencing and Hi-C data)	
Assembly metrics *		*Benchmark*
Consensus quality (QV)	67.1	*≥ 50*
*k*-mer completeness	100%	*≥ 95%*
BUSCO **	C:99.1%[S:98.6%,D:0.5%],F:0.2%,M:0.8%,n:5,286	*C ≥ 95%*
Percentage of assembly mapped to chromosomes	99.94%	*≥ 95%*
Sex chromosomes	Z chromosome	*localised homologous* *pairs*
Organelles	Mitochondrial genome assembled	*complete single alleles*
Raw data accessions		
PacificBiosciences SEQUEL II	ERR10798432	
Hi-C Illumina	ERR10802455	
Genome assembly		
Assembly accession	GCA_949091785.1	
*Accession of alternate haplotype*	GCA_948532955.1	
Span (Mb)	716.7	
Number of contigs	118	
Contig N50 length (Mb)	13.7	
Number of scaffolds	40	
Scaffold N50 length (Mb)	24.4	
Longest scaffold (Mb)	59.2	

* Assembly metric benchmarks are adapted from column VGP-2020 of “Table 1: Proposed standards and metrics for defining genome assembly quality” from ( Rhie *et al.*, 2021). ** BUSCO scores based on the lepidoptera_odb10 BUSCO set using v5.3.2. C = complete [S = single copy, D = duplicated], F = fragmented, M = missing, n = number of orthologues in comparison. A full set of BUSCO scores is available at https://blobtoolkit.genomehubs.org/view/ilPolLich1.1/dataset/CARWXJ01/busco.

**Figure 3.**
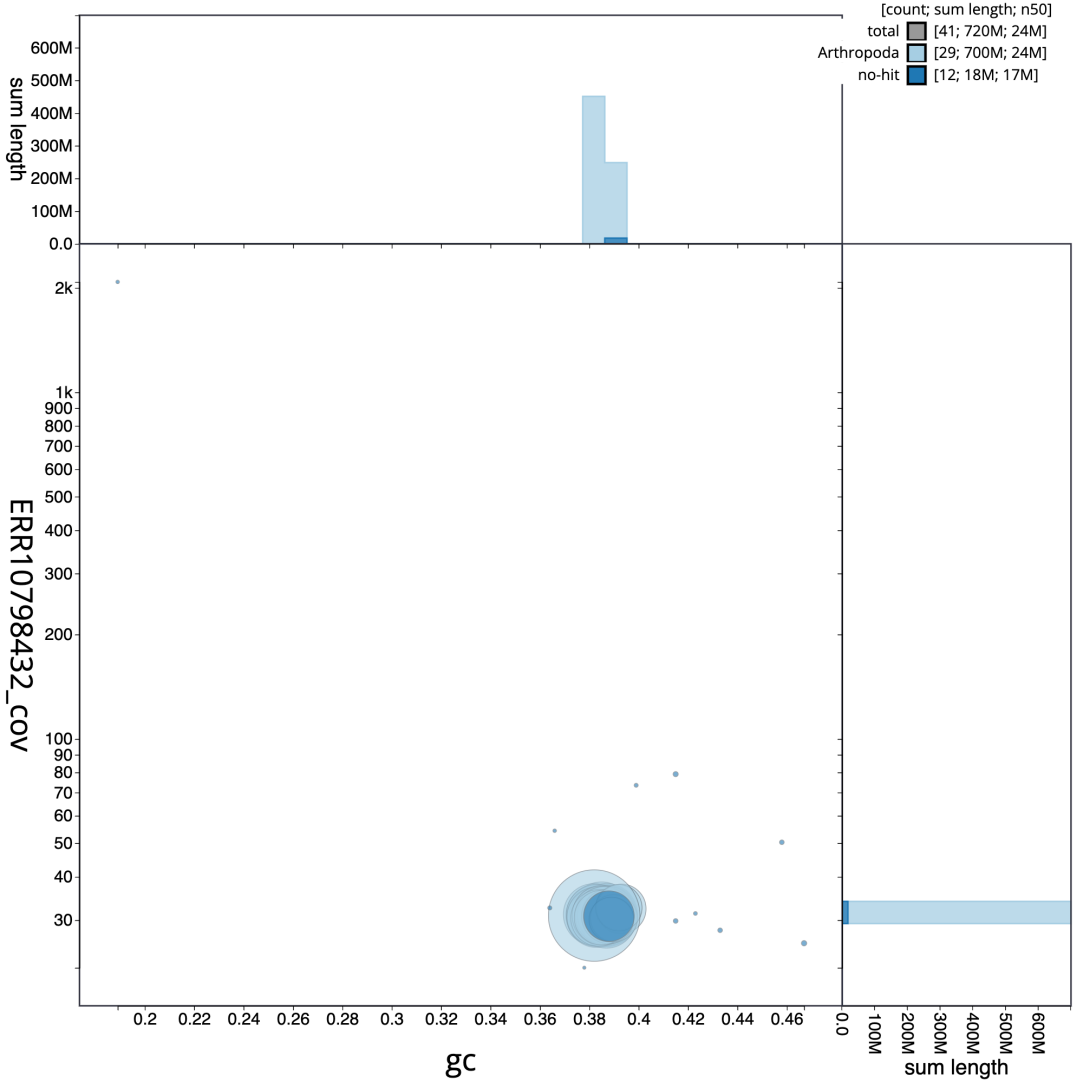
Genome assembly of *Polymixis lichenea*, ilPolLich1.1: BlobToolKit GC-coverage plot. Scaffolds are coloured by phylum. Circles are sized in proportion to scaffold length. Histograms show the distribution of scaffold length sum along each axis. An interactive version of this figure is available at https://blobtoolkit.genomehubs.org/view/ilPolLich1.1/dataset/CARWXJ01/blob.

**Figure 4.**
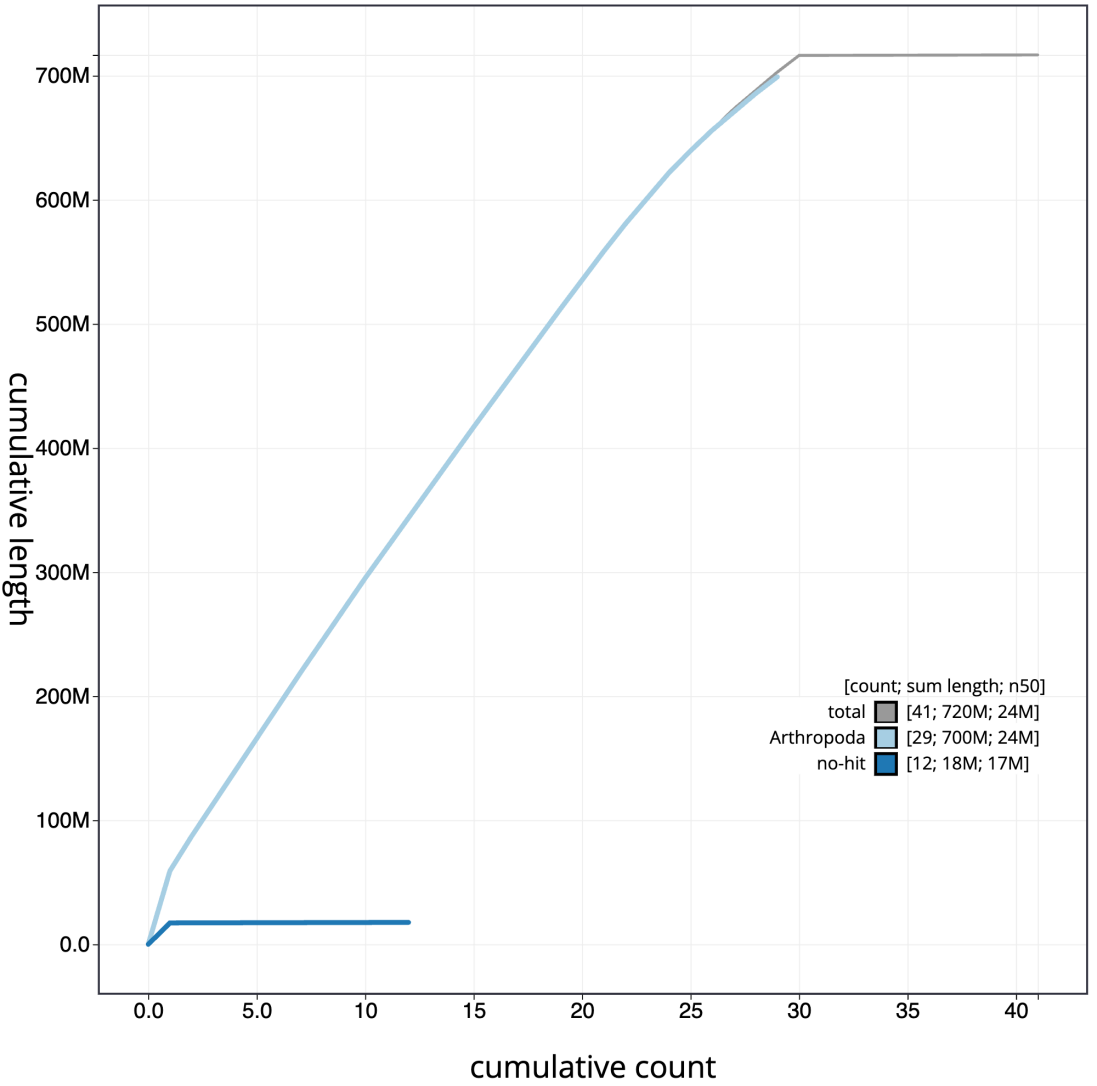
Genome assembly of *Polymixis lichenea*, ilPolLich1.1: BlobToolKit cumulative sequence plot. The grey line shows cumulative length for all scaffolds. Coloured lines show cumulative lengths of scaffolds assigned to each phylum using the buscogenes taxrule. An interactive version of this figure is available at https://blobtoolkit.genomehubs.org/view/ilPolLich1.1/dataset/CARWXJ01/cumulative.

**Figure 5.**
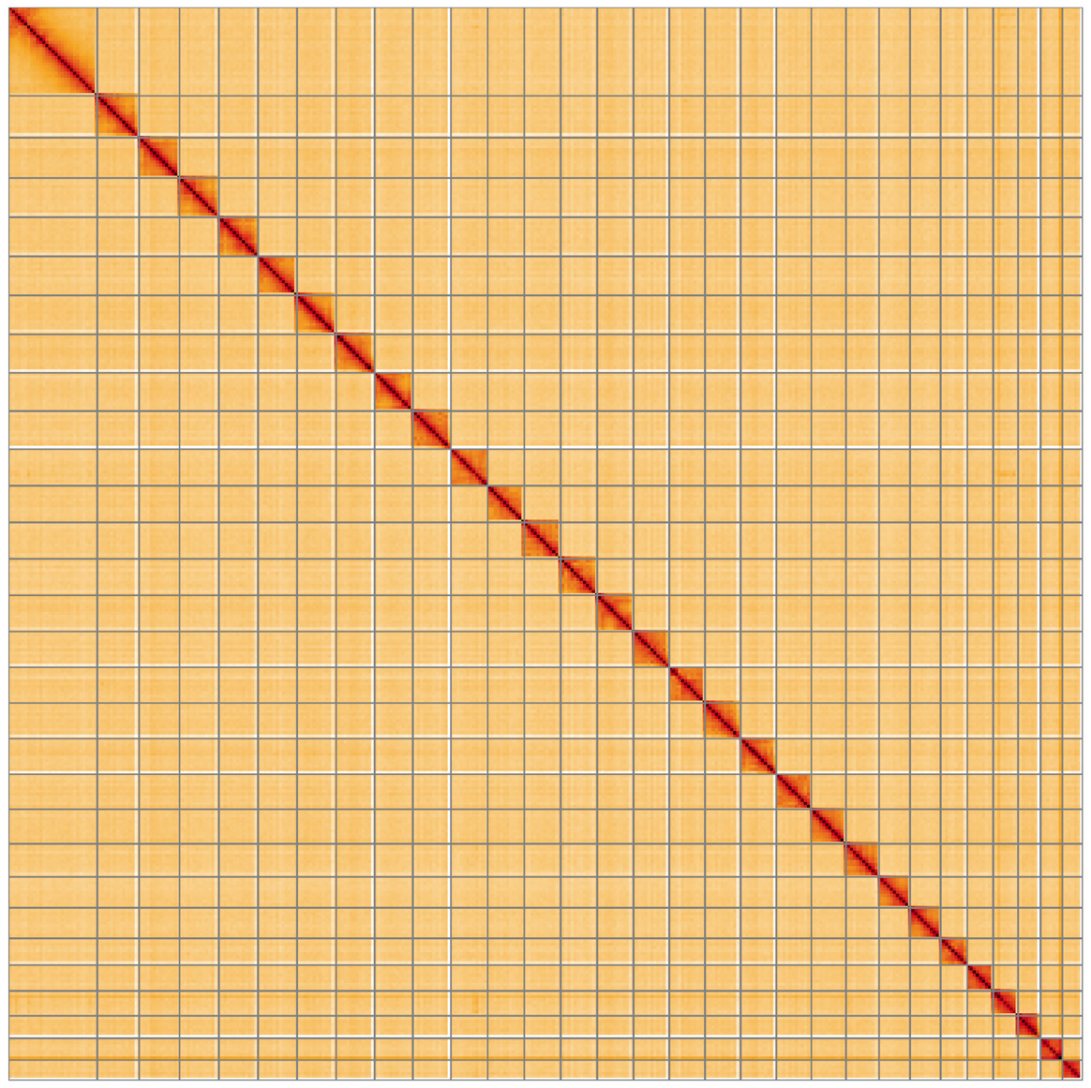
Genome assembly of *Polymixis lichenea*, ilPolLich1.1: Hi-C contact map of the ilPolLich1.1 assembly, visualised using HiGlass. Chromosomes are shown in order of size from left to right and top to bottom. An interactive version of this figure may be viewed at https://genome-note-higlass.tol.sanger.ac.uk/l/?d=W7Awu15tSs2lO-z-wTKsJA.

**Table 2.** Chromosomal pseudomolecules in the genome assembly of *Polymixis lichenea*, ilPolLich1.

INSDC accession	Chromosome	Length (Mb)	GC%
OX420932.1	1	27.92	38.5
OX420933.1	2	26.93	38.5
OX420934.1	3	26.16	38.5
OX420935.1	4	26.14	38.0
OX420936.1	5	26.12	38.5
OX420937.1	6	26.05	38.5
OX420938.1	7	25.65	38.5
OX420939.1	8	25.52	38.5
OX420940.1	9	25.42	38.5
OX420941.1	10	24.45	38.5
OX420942.1	11	24.41	38.5
OX420943.1	12	24.36	38.5
OX420944.1	13	24.3	38.5
OX420945.1	14	24.22	38.5
OX420947.1	16	23.86	38.5
OX420946.1	15	23.86	38.5
OX420948.1	17	23.8	38.5
OX420949.1	18	23.72	38.5
OX420950.1	19	23.37	38.5
OX420951.1	20	23.09	38.5
OX420952.1	21	22.13	38.5
OX420953.1	22	20.56	39.0
OX420954.1	23	20.45	38.5
OX420955.1	24	17.82	39.0
OX420956.1	25	17.27	39.0
OX420957.1	26	16.58	39.5
OX420958.1	27	15.03	39.0
OX420959.1	28	14.52	39.0
OX420960.1	29	13.42	39.0
OX420931.1	Z	59.17	38.0
OX420961.1	MT	0.02	19.0

The estimated Quality Value (QV) of the final assembly is 67.1 with *k*-mer completeness of 100%, and the assembly has a BUSCO v5.3.2 completeness of 99.1% (single = 98.6%, duplicated = 0.5%), using the lepidoptera_odb10 reference set ( *n* = 5,286).

Metadata for specimens, spectral estimates, sequencing runs, contaminants and pre-curation assembly statistics can be found at https://links.tol.sanger.ac.uk/species/1870252.

## Methods

### Sample acquisition and nucleic acid extraction

A male *Polymixis lichenea* (specimen ID NHMUK013698282, individual ilPolLich1) was collected from Restharrow Dunes National Nature Reserve, England, UK (latitude 51.27, longitude 1.38) on 2021-09-24. The specimen was collected and identified by David Lees (Natural History Museum) and dry frozen at –80°C.

The ilPolLich1 sample was prepared for DNA extraction at the Tree of Life laboratory, Wellcome Sanger Institute (WSI). The sample weighed and dissected on dry ice with tissue set aside for Hi-C sequencing. Head and thorax tissue was cryogenically disrupted to a fine powder using a Covaris cryoPREP Automated Dry Pulveriser, receiving multiple impacts. DNA was extracted at the WSI Scientific Operations core using the Qiagen MagAttract HMW DNA kit, according to the manufacturer’s instructions.

### Sequencing

Pacific Biosciences HiFi circular consensus DNA sequencing libraries were constructed according to the manufacturers’ instructions. DNA sequencing was performed by the Scientific Operations core at the WSI on a Pacific Biosciences SEQUEL II (HiFi) instrument. Hi-C data were also generated from remaining tissue of ilPolLich1 using the Arima2 kit and sequenced on the Illumina NovaSeq 6000 instrument.

### Genome assembly, curation and evaluation

Assembly was carried out with Hifiasm ( Cheng *et al.*, 2021) and haplotypic duplication was identified and removed with purge_dups ( Guan *et al.*, 2020). The assembly was then scaffolded with Hi-C data ( Rao *et al.*, 2014) using YaHS ( Zhou *et al.*, 2023). The assembly was checked for contamination and corrected as described previously ( Howe *et al.*, 2021). Manual curation was performed using HiGlass ( Kerpedjiev *et al.*, 2018) and Pretext ( Harry, 2022). The mitochondrial genome was assembled using MitoHiFi ( Uliano-Silva *et al.*, 2023), which runs MitoFinder ( Allio *et al.*, 2020) or MITOS ( Bernt *et al.*, 2013) and uses these annotations to select the final mitochondrial contig and to ensure the general quality of the sequence.

A Hi-C map for the final assembly was produced using bwa-mem2 ( Vasimuddin *et al.*, 2019) in the Cooler file format ( Abdennur & Mirny, 2020). To assess the assembly metrics, the *k*-mer completeness and QV consensus quality values were calculated in Merqury ( Rhie *et al.*, 2020). This work was done using Nextflow ( Di Tommaso *et al.*, 2017) DSL2 pipelines “sanger-tol/readmapping” ( Surana *et al.*, 2023a) and “sanger-tol/genomenote” ( Surana *et al.*, 2023b). The genome was analysed within the BlobToolKit environment ( Challis *et al.*, 2020) and BUSCO scores ( Manni *et al.*, 2021; Simão *et al.*, 2015) were calculated.

Table 3 contains a list of relevant software tool versions and sources.

**Table 3.** Software tools: versions and sources.

Software tool	Version	Source
BlobToolKit	4.1.5	https://github.com/blobtoolkit/blobtoolkit
BUSCO	5.3.2	https://gitlab.com/ezlab/busco
Hifiasm	0.16.1-r375	https://github.com/chhylp123/hifiasm
HiGlass	1.11.6	https://github.com/higlass/higlass
Merqury	MerquryFK	https://github.com/thegenemyers/MERQURY.FK
MitoHiFi	2	https://github.com/marcelauliano/MitoHiFi
PretextView	0.2	https://github.com/wtsi-hpag/PretextView
purge_dups	1.2.3	https://github.com/dfguan/purge_dups
sanger-tol/genomenote	v1.0	https://github.com/sanger-tol/genomenote
sanger-tol/readmapping	1.1.0	https://github.com/sanger-tol/readmapping/tree/1.1.0
YaHS	1.2a	https://github.com/c-zhou/yahs

### Wellcome Sanger Institute – Legal and Governance

The materials that have contributed to this genome note have been supplied by a Darwin Tree of Life Partner. The submission of materials by a Darwin Tree of Life Partner is subject to the **‘Darwin Tree of Life Project Sampling Code of Practice’**, which can be found in full on the Darwin Tree of Life website here. By agreeing with and signing up to the Sampling Code of Practice, the Darwin Tree of Life Partner agrees they will meet the legal and ethical requirements and standards set out within this document in respect of all samples acquired for, and supplied to, the Darwin Tree of Life Project. 

Further, the Wellcome Sanger Institute employs a process whereby due diligence is carried out proportionate to the nature of the materials themselves, and the circumstances under which they have been/are to be collected and provided for use. The purpose of this is to address and mitigate any potential legal and/or ethical implications of receipt and use of the materials as part of the research project, and to ensure that in doing so we align with best practice wherever possible. The overarching areas of consideration are:

• Ethical review of provenance and sourcing of the material

• Legality of collection, transfer and use (national and international) 

Each transfer of samples is further undertaken according to a Research Collaboration Agreement or Material Transfer Agreement entered into by the Darwin Tree of Life Partner, Genome Research Limited (operating as the Wellcome Sanger Institute), and in some circumstances other Darwin Tree of Life collaborators.

## Data Availability

European Nucleotide Archive: *Polymixis lichenea.* Accession number PRJEB59081; https://identifiers.org/ena.embl/PRJEB59081. ( Wellcome Sanger Institute, 2023) The genome sequence is released openly for reuse. The *Polymixis lichenea* genome sequencing initiative is part of the Darwin Tree of Life (DToL) project. All raw sequence data and the assembly have been deposited in INSDC databases. The genome will be annotated using available RNA-Seq data and presented through the Ensembl pipeline at the European Bioinformatics Institute. Raw data and assembly accession identifiers are reported in Table 1.

## Author information

Members of the Natural History Museum Genome Acquisition Lab are listed here: https://doi.org/10.5281/zenodo.4790042.

Members of the Darwin Tree of Life Barcoding collective are listed here: https://doi.org/10.5281/zenodo.4893703.

Members of the Wellcome Sanger Institute Tree of Life programme are listed here: https://doi.org/10.5281/zenodo.4783585.

Members of Wellcome Sanger Institute Scientific Operations: DNA Pipelines collective are listed here: https://doi.org/10.5281/zenodo.4790455.

Members of the Tree of Life Core Informatics collective are listed here: https://doi.org/10.5281/zenodo.5013541.

Members of the Darwin Tree of Life Consortium are listed here: https://doi.org/10.5281/zenodo.4783558.
