# Supplementary material for: The genome sequence of the Feathered Ranunculus, Polymixis lichenea (Hübner, 1813)
Source: Wellcome Open Res. 2023 Oct 12;8:444. [Version 1] doi: 10.12688/wellcomeopenres.20007.1 (PMC11303942; doi:10.12688/wellcomeopenres.20007.1)
Supplement: Peer review history

# Reviewer report: Reviewer response for version 1

**DOI:** 10.21956/wellcomeopenres.22153.r90255

**Published:** 2024-08-06

**Authors:** Tianzhu Xiong

**Affiliations:** 1 Cornell University, Ithaca, USA

The article presents a chromosome-level genome assembly of the Noctuid moth Polymixis lichenea. Methods based on PacBio and Hi-C are well described. I manually checked their Hi-C map and could not find obvious assembly errors. Based on other data presented here such as BUSCO, this is a high-quality reference genome.

Are sufficient details of methods and materials provided to allow replication by others?

Yes

Is the rationale for creating the dataset(s) clearly described?

Yes

Are the datasets clearly presented in a useable and accessible format?

Yes

Are the protocols appropriate and is the work technically sound?

Yes

Reviewer Expertise:

Evolutionary genomics

I confirm that I have read this submission and believe that I have an appropriate level of expertise to confirm that it is of an acceptable scientific standard.

# Reviewer report: Reviewer response for version 1

**DOI:** 10.21956/wellcomeopenres.22153.r68531

**Published:** 2023-11-03

**Authors:** Kuppusamy Sivasankaran

**Affiliations:** 1 Division of Taxonomy and Biodiversity, Entomology Research Institute, Loyola College, Chennai, Tamil Nadu, India

I appreciate the authors for assembling the *Polymixis lichenea* whole genome sequence. The authors have used the proper software for assembly and annotation of the genome.

**Comments:**

1. Page no. 3 - In the second paragraph, the second line “is a moderately sized noctuid” can be changed to “is a moderately sized Noctuid”
2. Page no. 3 - The second paragraph's third line can be changed to “Forewing greenish tint, finely dotted with grey”.
3. Page no. 3 - sixth paragraph second column 4 ^th^ line there is a typo error. place *P. lichenea i*n the….alphabet ‘i' is in italics. It can be changed to a normal letter.

 

**Question:**

- Why do authors not annotate the genome of *P. lichenea*? In the genome annotation authors could get the number of coding genes, non-coding genes, and transcript genes.

Overall, the manuscript can be accepted for indexing.

Are sufficient details of methods and materials provided to allow replication by others?

Yes

Is the rationale for creating the dataset(s) clearly described?

Yes

Are the datasets clearly presented in a useable and accessible format?

Yes

Are the protocols appropriate and is the work technically sound?

Yes

Reviewer Expertise:

Mitochondrial genome sequencing for phylogenetic study

I confirm that I have read this submission and believe that I have an appropriate level of expertise to confirm that it is of an acceptable scientific standard.
